# Targets and Gene Therapy of ALS (Part 1)

**DOI:** 10.3390/ijms26094063

**Published:** 2025-04-25

**Authors:** Olga Shiryaeva, Christina Tolochko, Tatiana Alekseeva, Vyacheslav Dyachuk

**Affiliations:** Almazov Federal Medical Research Centre, 197341 Saint Petersburg, Russia; shiryaeva_os@almazovcentre.ru (O.S.); tolochko_ka@almazovcentre.ru (C.T.); alekseeva_tm@almazovcentre.ru (T.A.)

**Keywords:** SOD1, *C9orf72*, FUS, TARDBP, gene therapy of ALS, ALS pathogenesis, RNA interference, antisense oligonucleotides (ASO), CRISPR/Cas9

## Abstract

Amyotrophic lateral sclerosis (ALS) is a neurodegenerative disease characterized by the selective death of motor neurons, which causes muscle atrophy. Genetic forms of ALS are recorded only in 10% of cases. However, over the past decade, studies in genetics have substantially contributed to our understanding of the molecular mechanisms underlying ALS. The identification of key mutations such as *SOD1*, *C9orf72*, *FUS*, and *TARDBP* has led to the development of targeted therapy that is gradually being introduced into clinical trials, opening up a broad range of opportunities for correcting these mutations. In this review, we aimed to present an extensive overview of the currently known mechanisms of motor neuron degeneration associated with mutations in these genes and also the gene therapy methods for inhibiting the expression of their mutant proteins. Among these, antisense oligonucleotides, RNA interference (siRNA and miRNA), and gene-editing (CRISPR/Cas9) methods are of particular interest. Each has shown its efficacy in animal models when targeting mutant genes, whereas some of them have proven to be efficient in human clinical trials.

## 1. Introduction

Amyotrophic lateral sclerosis (ALS) is a neurodegenerative disease characterized by the selective death of motor neurons leading to muscle atrophy. Sporadic ALS occurs in 90% of cases, while hereditary mutations make up only 10%. Despite this discrepancy, studies on ALS gene therapy are actively underway. Recent data have shown major implications of ALS gene therapy not only for patients who suffer from the genetic form of ALS but also for those with the sporadic form. In an examination of 2340 patients with sporadic ALS, a pathogenic variant of *C9orf72* was recorded in 13% [1]. These data show that some patients with sporadic cases may also need gene therapy against mutant proteins.

Our review is focused on studies of the effects of ALS gene therapy targeting the known mutations, specifically in *SOD1*, *C9orf72*, *FUS*, and *TARDBP*. Mutations in these genes lead to disturbances of functions essential for cell life by affecting the levels of certain mRNAs [2,3,4,5,6], the formation of protein aggregates [7,8], and disruption of autophagy [9,10,11], DNA damage repair [12,13,14], and synaptic transmission [15]. Gene therapy approaches including antisense oligonucleotides, RNA interference methods (siRNA and miRNA), and the genome-editing method (CRISPR/Cas9) have demonstrated muted cytotoxic effects of these mutant genes in animal models. In clinical studies, the effect of antisense oligonucleotides has been studied more in detail [16,17,18,19], and some of them are still actively conducted today [20]. Drugs aimed at RNA interference are only entering clinical trials. In 2024, the first drug RAG-17 entered into phase 1 clinical trials. Currently, gene-editing methods based on the CRISPR/Cas9 system successfully correct mutations in cell cultures and mouse models [21,22].

## 2. Familial Forms of ALS

### 2.1. Mutations in the SOD1 Gene

Mutations in the *SOD1* gene account for approximately 15–20% of cases of hereditary ALS in various populations [23]. These are the second most prevalent after *C9orf72* mutations. More than 170 various kinds of mutations in this gene have been documented as point mutations, deletions, and other types of modifications (https://alsod.ac.uk/). The most common among them are D90A, A4V, and G93A [24]. The main pathogenetic theory associated with mutations in the SOD1 gene, on which all therapeutic approaches are currently focused, is protein aggregate formation [25]. Variants of aggregates can be formed due to disturbances in metal binding, structural stability, and protein aggregation. In cells, two systems are distinguished to prevent the formation of damaged proteins: ubiquitin–proteasome (UPS) and autophagy–lysosome (ALP) systems [26]. However, the resulting mutant SOD1 aggregates are capable of blocking UPS; thus, the number of aggregates in cells will increase. Improperly folded SOD1 molecules are observed in nuclei of astrocytes, microglia, oligodendrocytes, and motor neurons in patients with ALS, both with and without *SOD1* mutations [7]. Such protein aggregates cannot perform their functions and provide increased toxic activity [27,28]. One of the principles of this toxicity is the conformational change in the Bcl-2 protein. Under normal conditions, Bcl-2 protects cells by blocking the release of pro-apoptotic molecules from the mitochondria. However, under interaction with the modified SOD1 protein, this protective function is disrupted, which induces the release of cytochrome *c* and activates apoptosis [29,30]. Bsl-2 family proteins are also involved in mitochondrial respiration and oxidative metabolism. Pro-apoptotic Bsl-2 family proteins contribute to glycolysis enhancement and activation of the pentose phosphate pathway during phosphorylation, limiting the efficacy of mitochondrial respiration [31]. With the long-term dominance of such metabolic processes, when glycolysis becomes the major source of energy, mitochondrial decompensation may occur, which leads to the inability of cells to produce ATP.

Furthermore, SOD1 aggregates can disrupt axonal transport through specific interactions with neurofilament light chain mRNA and the dynein–dynactin complex [32] and hamper the supply of essential nutrients to neuronal endings. This is of particular importance, because motor neurons have long axons and the disruption of transport processes can cause functional disorders and the atrophy of these cells.

Impaired gene expression and mRNA regulation also play an important role in the pathogenesis of ALS. Under normal conditions, SOD1 is able to change its localization from predominantly cytoplasmic to predominantly nuclear, where it promotes the activation of genes involved in cellular resistance to ROS in response to oxidative stress [33]. Mutations lead to the decreased activation of genes responsible for protection against oxidative stress, which leads to increased levels of reactive oxygen species (ROS), which cause cell damage.

SOD1 is also involved in the regulation of certain genes, and its action may affect the level of synthesis of certain mRNAs associated with the regulation of various cellular processes such as the cell cycle, apoptosis, and stress response. Studies showed a decrease in the expression of the mRNA of vascular endothelial growth factor (VEGF) in the spinal cords of mice with the G93A mutation of the *SOD1* gene in early stages of the disease. VEGF exhibits neuroprotective properties, and the loss of its expression may lead to a phenotype similar to ALS even with the lack of *SOD1* mutations [2,3].

It was also shown that the A4V SOD1 mutant, by binding to the 3′UTR of NF-L mRNA, destabilizes it and leads to a decrease in NF-L protein synthesis. This reduces the proportion of NF-L compared to other subunits of NF and contributes to the aggregation of NF in the perikaryon and proximal part of the axon. Such aggregates can disrupt axonal transport and contribute to neuronal death [4]. A reduced expression of mRNAs that encode neurofilament subunits (NF-L, NF-M, and NF-H) was also detected in motor neurons in cases with familial ALS [34].

### 2.2. Mutations in the TARDBP Gene

Mutations in the *TARDBP* gene have been recorded in 5% of familial ALS cases and are the cause of about 1% of sporadic ALS [35,36,37]. To date, about 70 mutations in this gene are known.

*TARDBP* encodes the transactive response (TAR) DNA-binding protein of 43 kDa (TDP-43), which was first identified in 1995 as a suppressor of HIV-1 gene expression [38]. In 2006, it was recognized as a key component of insoluble ubiquitinated inclusions found in 97% of cases of patients with ALS [36,39,40,41]. TDP-43 is usually located in the nucleus, but it can also move between the nucleus and the cytoplasm [42]. In ALS, the level of TDP-43 in the cytoplasm is observed to increase, which leads to the formation of cytoplasmic inclusions that disrupt cell function [43]. Moreover, the mitochondrial localization of TDP-43 ALS may lead to mitochondrial dysfunction due to the accumulation of aggregates that can impair the translation of mRNA encoding the subunits of the respiratory complex [44].

The TDP-43 protein performs essential functions in the cell, including the transcription and splicing of mRNA, being involved in the biosynthesis of microRNAs, processing of non-coding RNAs, stabilization of mRNA in the cytoplasm, regulation of translation, and formation of stress granules and ribonucleoprotein transport granules [45,46,47,48].

It regulates mRNA processing, RNA export, and RNA stability due to the presence of highly conserved recognition motifs (RRM1 and RRM2 domain), required for binding to various RNA/DNA molecules [5,49]. These domains allow TDP-43 to interact with thousands of mRNA transcripts, including its own mRNA, which is important for the autoregulation of its concentration and, probably, solubility [50]. In ALS, mutations in the region of recognition motifs are produced that can disrupt the binding of TDP-43 to nucleic acids, which may play a role in the loss of the normal regulation of gene expression and mRNA stability [51]. The accumulation of TDP-43 in the cytoplasm also contributes to the disruption of translation processes. These data were obtained from neuroblastoma cells [51].

The highest levels of TDP-43 are observed in the euchromatin domains of neurons where transcription occurs, but its exact role in these processes has not yet been sufficiently studied [52].

The TDP-43 protein regulates the expression of the ATG7 protein and is thus involved in autophagy regulation [9]. During the initiation of autophagy, the ATG7 protein plays a key role by functioning as an E-1 enzyme for ubiquitin-like proteins including ATG12 and ATG8. After binding to the latter, ATG7 catalyzes their activation, which allows the ubiquitin-like proteins to be involved in the assembly of autophagosomes. After the activation, ATG7 transfers ATG12 and ATG8 to the E-2 enzyme. ALS-associated mutations in the *UBQLN2* gene cause a disturbance of autophagy and an increase in the total level of TDP-43, which contributes to its aggregation and causes cytotoxic functions to appear [53].

TDP-43 plays a major role in the regulation of non-coding RNAs, including microRNAs (miRNAs) involved in gene expression regulation. The Drosha protein complex associated with TDP-43 performs the initial cleavage of long primary microRNAs (pri-miRNAs) into shorter microRNA precursors. TDP-43 binds directly to these pri-miRNAs, which contributes to their correct processing and formation of functional pre-miRNAs [47]. The tissues where TDP-43 level is observed to decrease show abnormalities in the expression of various microRNAs. Studies indicate that the inhibition of TDP-43 in HeLa cells, rodent neurons, and human neurons resulted in significant changes in the expression profile of several miRNAs [50,54].

TDP-43 is involved in alternative splicing, thereby influencing the expression of its own transcript or the statmin-2 transcript that binds to the α- and β-tubulin composing microtubules. A reduction in statmin-2 transcription leads to the breakdown of microtubules, which causes the disturbance of axonal transport and contributes to motor neuron degeneration [55,56,57]. TDP-43 also binds to other transcripts, including pre-mRNAs and miRNA precursors [54]. This binding plays a major role in the regulation of their stability, splicing, and transport, as well as in the process of RNA metabolism in general. Furthermore, TDP-43 is involved in the repair of damaged DNA sites through the non-homologous end joining (NHEJ) mechanism, which can be disrupted in ALS [12,13].

### 2.3. Mutations in the FUS Gene

Mutations in the *FUS* gene occur in about 4% of familial cases and in less than 2% of patients with sporadic ALS. A total of more than 120 known mutations in this gene are known to date [58]. Scientists first demonstrated that *FUS* mutations cause ALS in 2009 [59]. The *FUS* gene encodes the RNA-binding FUS protein that shows structural resemblance to the TDP-43 protein. In normally functioning cells, FUS has a nuclear localization where it plays an important role in the transcription, splicing, RNA stabilization, and DNA repair processes [60]. In ALS, an increase in this protein is observed in the cytoplasm [61].

The FUS protein, as well as TDP-43, plays a major role in DNA repair, showing the ability to restore it both by homologous recombination and by NHEJ. The mutant FUS protein is capable of restoring NHEJ to a greater extent [14]. With the lack of the FUS protein, the repair was decreased to 50% [62]. In familial ALS, patients with the R521C or P525L *FUS* mutations showed increased levels of DNA damage compared to the control group, which was also demonstrated for primary cultured neurons [62].

FUS is involved in the formation of stress granules (SGs), which are structures consisting of mRNA and proteins required for the initiation of translation. SGs are formed in response to oxidative stress, viral infections, or nutrient deficiencies. Under such conditions, they suppress the translation of most proteins, while simultaneously enhancing the synthesis of those that help cells cope with stress [63]. The mutant variant of FUS, while accumulating in the cytoplasm, can bind to the components of stress granules, leading to an increase in their size, number, and the aggregation of proteins inside of them. This interaction disrupts the normal cell response to stress, as was also shown in a study on human cells with the P525L mutation [8].

FUS is involved in the regulation of the transcription of genes influencing the activity of RNA polymerase II, which is responsible for mRNA synthesis and, therefore, has an effect on cell processes such as growth, differentiation, and stress response [6]. In addition, FUS can inhibit the activity of RNA polymerase III, which synthesizes small RNAs, e.g., tRNA and rRNA [64]. The inhibition of this enzyme can play a key role in maintaining the proper balance between different RNAs and in preventing the excessive synthesis of certain molecules, which is important for maintaining cellular homeostasis. In the context of ALS mutations, FUS exerts rather an indirect effect on the expression mediated by pre-mRNA processing [61,65].

FUS is involved in regulating the activity of NMDA receptors, which play a key role in synaptic transmission. Furthermore, FUS is localized in the transport granules responsible for the transport of proteins and other molecules to synapses. However, one study [15] showed that the postnatal knockout of FUS in a mouse model did not cause the motor neuron degeneration phenotypes and synaptic failure observed in mice carrying the ALS FUS mutation, implying that altered levels of endogenous (wild-type) FUS does not contribute to motor neuron death. Rather, the presence of ALS-associated FUS mutants, R521C and P525L, is the cause of motor neuron death.

In 2010, researchers paid attention, for the first time, to the mitochondrial disorganization associated with the P525L mutation in the *FUS* gene [66]. Six years later, in 2016, it was found that the FUS protein is important for the activation of the GSK-3β enzyme. The latter is involved in the regulation of the interaction between the VAPB and PTPIP51 proteins that form contact sites between the endoplasmic reticulum and mitochondria. The cytoplasmic contacts created through such an interaction are essential for the adequate uptake of calcium by the mitochondria after its release from endoplasmic reticulum (ER) cisterns. With the release of Ca^2+^, the VAPB protein is bound to PTPIP51, which contributes to the formation of stable contacts between these organelles, providing efficient signal transmission and the maintenance of calcium homeostasis. The aggregation of the FUS protein induces the activation of GSK-3β and the disruption of the VAPB–PTPIP51 interaction in ALS. These changes also negatively affect mitochondria functions [67]. In 2020, it was found that mutations in the *FUS* gene such as R521C or its overexpression lead to the dysfunction of type I NADH dehydrogenase. This results from FUS binding to the mRNA of mitochondrial proteins, which disrupts their translation. Studies on the use of mitotrackers have shown a number of disorders in the mitochondrial network—an irregular arrangement of mitochondria, a shortening of their structure, and an increase in the level of mitochondrial ROS—that were probably associated with a moderate decrease in the RCC protein level, which belongs to the respiratory chain complex [68].

### 2.4. Mutations in the C9orf72 Gene

A mutation in the *C9orf72* gene was first identified in 2011. As a result of this mutation, the GGGGCC hexanucleotide repeat is expanded, which reduces the total number of *C9orf72* transcripts and causes toxic products to form in the cell [69]. This mutation is most widely distributed in Europe and North America and may account for up to 46.0% of familial and 21.1% of sporadic ALS [70].

The *C9orf72* protein is mainly localized in the nucleus. Its major functions include in endosomal transport, the regulation of autophagy, the modulation of inflammatory processes, and the activation of microglia.

The *C9orf72* protein interacts with the following Rab family proteins that play a key role in autophagy, endosomal transport, and other cellular processes: Rab1, Rab5, Rab7, and Rab11. Among them, Rab7 and Rab11 are localized in motor neurons. In ALS patients with the *C9orf72* mutation, an increase has been observed in the number of cells with *C9orf72* colocalization with Rab7 or Rab11 compared to the control group, which indicates the disruption of endosomal transport regulation in ALS patients [10].

*C9orf72* is involved in autophagy regulation. As was demonstrated in a study in 2014, *C9orf72* is colocalized with DsRed-tagged microtubule-associated protein 1 light chain 3 (DsRed-LC3), which is one of the highly specific markers of autophagosomes [10]. In 2018, the mechanism of autophagy promotion by *C9orf72* through regulating the functioning of the Rab1a protein was studied in detail. The importance of Rab1a lies in its ability to translocate the ULK1 complex, a key regulator of autophagy initiation, to the phagophore. However, the repeat expansion in the *C9orf72* gene in ALS disrupts this interaction, which causes a decrease in autophagy efficiency [11].

The study of the role of *C9orf72* in immune system activation is currently underway. To date, it remains unknown whether *C9orf72* mutations are a trigger of microglia activation or the latter is a consequence of neurodegeneration. ALS patients with the *C9orf72* mutation show an increased expression of genes such as Iba1 and CD86, which indicates an activated state of microglia [71]. Iba1 is a marker of microglia activation, and CD86 is involved in the activation of T cells, which suggests an increased immune response in brain tissue. Furthermore, the increased production of pro-inflammatory cytokine and ROS are also noted [72].

## 3. Gene Therapy Methods

**Figure 1:**
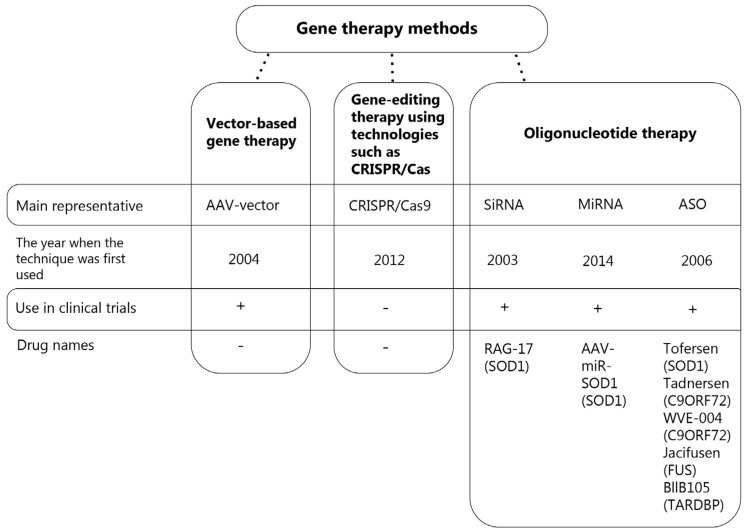
Gene therapy methods in the treatment of ALS.

Since the properties of mutant genes involved in ALS pathogenesis have not yet been fully elucidated, various gene therapy approaches to eliminating the toxicity of these genes are currently studied. The areas of gene therapy aimed at the inhibition of target gene expression by influencing mRNA or using genome-editing methods are particularly actively developing. The major areas of interest are listed in Figure 1.

### 3.1. Antisense Oligonucleotide Therapy

Among oligonucleotides, the most widespread are antisense ones, which account for approximately 65% of the total number of oligonucleotides [73]. These short chains, ranging in length from 15 to 30 nucleotides (nt), are complementary to certain mRNA molecules. The process of antisense oligonucleotide therapy begins with the selection of a specific mRNA to be suppressed. Then, a complementary oligonucleotide is synthesized that is capable of binding exclusively to the target mRNA molecule.

For the treatment of CNS disorders, oligonucleotides are delivered intrathecally, from where they enter cells through endocytosis. However, achieving the required selectivity is not always possible. To improve the accuracy of delivery, viral and non-viral vectors are used that direct the oligonucleotides to the target cells.

**Figure 2:**
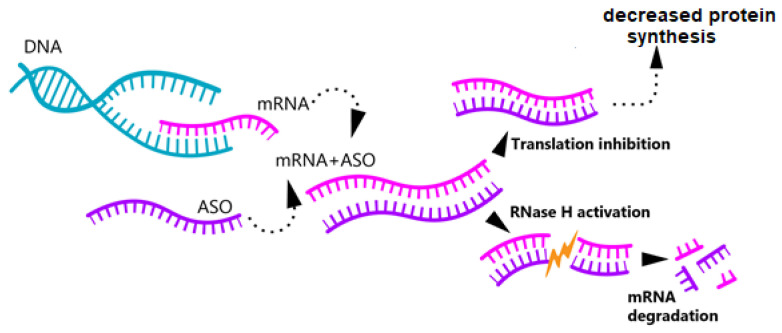
Mechanism of ASO action. After administration, the antisense oligonucleotide (ASO) binds complementarily to a single-stranded mRNA, which may lead to: (1) Activation of RNase H, contributing to chain breakdown and subsequent degradation; (2) Translation inhibition.

Upon delivery, the oligonucleotide binds to the target mRNA via base complementarity. As a result, one of two possible events occurs: mRNA degradation is triggered if the oligonucleotide activates RNase H, an enzyme that promotes the breakdown of the RNA–DNA complex, or the binding to the target mRNA blocks its translation (Figure 2) [73,74].

#### 3.1.1. *SOD1*

Antisense oligonucleotides were first applied to the treatment of ALS in 2006 using a strain of Sprague–Dawley rats with the *SOD1*^G93A^ gene mutation. The therapy was carried out with the oligonucleotide SOD r/h333611 (referred to as tofersen) targeted at SOD1. The oligonucleotide was administered intrathecally at a dose of 100 μg/day continuously for 28 days using an osmotic capsule. As a result, this infusion allowed for slowing down the progression of the disease and increased the survival of the animals by an average of 10 ± 7 days [75]. Furthermore, the introduction of antisense oligonucleotides decreased the levels of mRNAs in human fibroblasts with the *SOD1*^A4V^ mutation [75]. These promising data from preclinical studies became the basis for moving tofersen on to phase 1 clinical trials in 2013. A randomized placebo-controlled trial involved 21 people with confirmed mutations in the *SOD1* gene. The drug was administered intrathecally for 11.5 h at doses increased from 0.15 to 3 mg. Tofersen demonstrated good tolerability, safety, and effectiveness with intrathecal administration, which suggests its significant potential in ALS therapy [16].

Since 2018, preclinical studies of more potent antisense oligonucleotides have appeared in the scientific literature, which, like tofersen, substantially improve the levels of SOD1 mRNA and NF-L chains. However, these new oligonucleotides have also demonstrated additional potential that contributes to maintaining the state of neuromuscular synapses, delaying the onset of disease, and increasing the life expectancy of rats and mice [76]. These promising results highlight the need for further study of new forms of antisense oligonucleotides and their application in clinical trials.

##### Clinical Studies

After 2016, tofersen became a participant in phases 1–2 trials with 50 people involved. In the study, the drug was administered at doses from 20 to 100 mg. Each participant was randomly assigned to receive five doses of tofersen or a placebo that consisted of intrathecal injections for 12 weeks. The test results showed insignificant side effects that were mainly associated with lumbar puncture. There was a decrease in the level of SOD1 protein in the cerebrospinal fluid (CSF) to 36% depending on the dose; a decrease in the concentration of phosphorylated neurofilament heavy (NF-H) chains and neurofilament light (NF-L) chains was also recorded from both plasma and CSF [17]. These data confirmed the researchers’ expectations that tofersen might have a pronounced effect by reducing the mutant protein and markers of motor neuron degeneration. However, as was demonstrated at the phase 3 VALOR placebo-controlled trial, which was conducted from March 2019 to July 2021 and involved more than 100 people, despite the observed decrease in the level of SOD1 protein in CSF and the decrease in the concentration of NF-L chains of plasma, neither the tofersen group nor the control group that received the placebo showed any clinically significant improvement [18]. These results indicate the insufficiency of data on the widespread use of this therapy method and emphasize the importance of further studies, especially at earlier stages of the disease. To this end, the Open Label Extension (OLE) phase of the VALOR study was launched, allowing all participants who completed the main part of the study to access tofersen. This decision allowed us to evaluate the long-term safety and efficacy of the drug in both active and placebo-treated patients. The first OLE milestone was planned for 52 weeks after the start of treatment, and the next milestone is expected in 3.5 years. At the time of writing, the results of this study have not yet been published, but in 2023, the FDA approved tofersen for the treatment of ALS, and in May 2024, the drug received approval from the European Medicines Agency (EMA).

Tofersen is currently being studied in asymptomatic patients with a confirmed SOD1 mutation (NCT04856982) and in patients with a genetic risk for ALS-Pre-fALS (NCT00317616). Both of these studies were the first of their kind and are aimed at using gene therapy in asymptomatic ALS patients. This opens up new prospects for early intervention and the potential slowing of disease progression.

#### 3.1.2. *C9orf72*

In 2013, three studies assessed the effects of antisense oligonucleotides against hexanucleotide repeats in the *C9orf72* gene in patients’ fibroblasts [77,78,79]. In all cases, antisense oligonucleotides acted by the RNase H activation mechanism that provided the degradation of the repeat-containing “toxic” RNA transcripts. As a result, the number of cells with toxic foci significantly reduced, while RNA remained that did not contain hexanucleotide repeats.

Since 2016, researchers have switched to mouse models. In the first experiment, mice were injected a single dose of antisense oligonucleotides, which within two weeks resulted in a decrease in the level of repeat-containing *C9orf72* RNA in the cortex and spinal cord to 23 and 12% of control levels [80].

In February 2021, a study was published on new stereopure oligonucleotides complementary to *C9orf72* mRNAs containing “toxic” repeats [81]. One of these oligonucleotides was the drug WVE-004. During a study on a mouse model, there was an effective depletion of pathogenic mRNAs, while the expression of normal protein was not impaired [82].

##### Clinical Studies

Based on the successful results achieved with the use of antisense oligonucleotides on cell models and in experimental studies on mice, a phase 1 clinical trial of the drug Tadnersen was initiated that involved 106 people. The study began in 2018 and was completed by the end of 2021, though the results of the trial have not been officially published to date. The drug was administered intrathecally five times over eight months and, despite good tolerability among patients, did not show clinically significant effects, which resulted in the decision to stop its further development.

Having obtained encouraging data when studying new stereopure oligonucleotides, the scientists conducted a second clinical trial from 2021 to 2023. However, the novel drug also showed no clinical improvement, and the development of WVE-004 was discontinued. Thus, despite innovative approaches, the search for effective treatment using drugs based on the degradation of toxic mRNAs is still underway.

To date, among all antisense drugs, afinersen has attracted special attention. Although there are no detailed clinical studies yet, the drug was tested on a 60-year-old man who received eight injections with increasing doses. However, no negative reactions were recorded then, and the level of *C9orf72* with pathological repeats in CSF decreased by 80% [20].

#### 3.1.3. *FUS*

The drug Jasifusen, developed against the *FUS* gene mutation, was subjected to preclinical testing on a mouse model. The results were impressive: a single Jasifusen injection provided the complete prevention of muscle denervation and microgliosis for more than four months. However, six months later, when the concentration of the drug in the body reduced, the level of mutant *FUS* began to increase again [19].

Currently, researchers are developing modification of antisense oligonucleotides targeting the mutant FUS protein. The ASO with the most promising approach is allele-selective. These antisense oligonucleotides are designed to act exclusively in the mutant allele, while not affecting the normal one, which allows for retaining the functions of the healthy protein. The ongoing research has already yielded encouraging results on human fibroblasts, thus opening up new opportunities for further experiments [83].

##### Clinical Studies

After these first encouraging data, Jasifusen was tested on a 25-year-old woman who suffered from ALS. For 10 months, the patient received 12 injections with a gradually increasing dosage from 20 to 120 mg. The drug was well tolerated and did not cause side effects, which gave hope for a positive outcome. However, the progression of the disease did not slow down, and the patient died a year after the therapy started.

It is impossible to make an unambiguous conclusion as to whether the suppression of *FUS* has any effect on the progression of the disease when based solely on the treatment of a single patient. However, immunohistochemical staining, performed in cell line studies, showed the predominantly nuclear localization of *FU*S in spinal tissue, while numerous abnormal cytoplasmic and intracellular aggregates inside neurons were found in untreated samples [19].

Since 2021, the drug Jacifusen has been involved in a long-term phase 1–3 study aimed at evaluating its efficacy, safety, pharmacokinetics, and pharmacodynamics. The results of this study have not been reported to date, and the completion is scheduled for 2026.

#### 3.1.4. *TARDBP* and *ATXN2*

To date, no antisense oligonucleotides have been developed that directly target TARDBP gene mutations due to the fundamental role of the TDP-43 protein, and an approach involving the complete reduction in its levels is excluded.

The ataxin-2 (Atx2) protein, encoded by the ATXN2 gene, has multiple functions in the cell due to its structure and interactions with other molecules. It contains two globular domains near the N-terminal end. The first domain includes a clathrin-containing region that allows for the interaction with the trans-compartment of the Golgi complex and is necessary for processes associated with transport and sorting of proteins within the cell. The second domain serves to interact with the ER, indicating its involvement in protein synthesis and modification [84]. However, the exact functions of ataxin-2 are not fully understood; it has been shown that Atx2 is involved in the regulation of mRNA translation through interactions with PBP1. This interaction may influence mRNA stability and ribosome accessibility and the formation of SGs and processing bodies (p-bodies), which regulate RNA and the cell’s response to stress conditions. Two studies using Drosophila have shown that TDP-43, when forming aggregates, colocalizes with ATXN2 aggregates in the cytoplasm and interacts in an RNA-dependent manner [85].

TDP-43 contains two RRM (RNA Recognition Motif) domains that are required for RNA binding. Mutations in these domains result in the loss of the ability of TDP-43 to interact with ATXN2. Furthermore, the treatment of cell lysates with RNase resulted in the abolition of the interaction between TDP-43 and ATXN2 [85]. Thus, there is a high probability that these proteins bind to the same mRNAs, and while this ability to bind to RNA is maintained, TDP-43 aggregation occurs in the cytoplasm associated with ATXN2, and polyQ extensions in ATXN2 enhance this association and increase the stability of ATXN2, increasing TDP-43 aggregation [84]. In a mouse model, reducing Atxn2 protein levels significantly increased survival by reducing TDP-43 toxicity. In transgenic mice with abnormal TDP-43 expression (TDP-43 Tg/Tg Atxn2+/+), all animals died within 29 days. In contrast, the TDP-43 Tg/Tg Atxn2−/− group survived for over 300 days. Importantly, Atxn2 reduction did not affect mRNA expression levels of the human TDP-43 transgene or total TDP-43 levels in brain tissue. This rules out the possibility that the beneficial effect is solely due to a reduction in the toxic protein. The effect of Atxn2 reduction was also shown to be mediated through the modulation of stress granules. Atxn2-deficient mice showed a significant reduction in the proportion of cells containing TDP-43 stress granules, suggesting a reduced propensity for TDP-43 to be incorporated into pathological aggregates. This was confirmed by immunohistochemistry: The number of neurons with inclusion bodies positive for phosphorylated TDP-43 (pTDP-43 Ab1 and Ab2 antibodies) was reduced by 75% and 45%, respectively, in TDP-43 Tg/Tg Atxn2−/− mice, and ASOs targeting Atxn2 expression injected into the cerebral ventricles of neonatal mice resulted in a 77% reduction in Atxn2 mRNA levels by day 21 of life. These interventions did not alter transgenic TDP-43 expression but did result in a significant increase in survival (by an average of 35%) and improved motor performance [86].

### 3.2. Therapy with Small Interfering RNAs and miRNAs

**Figure 3:**
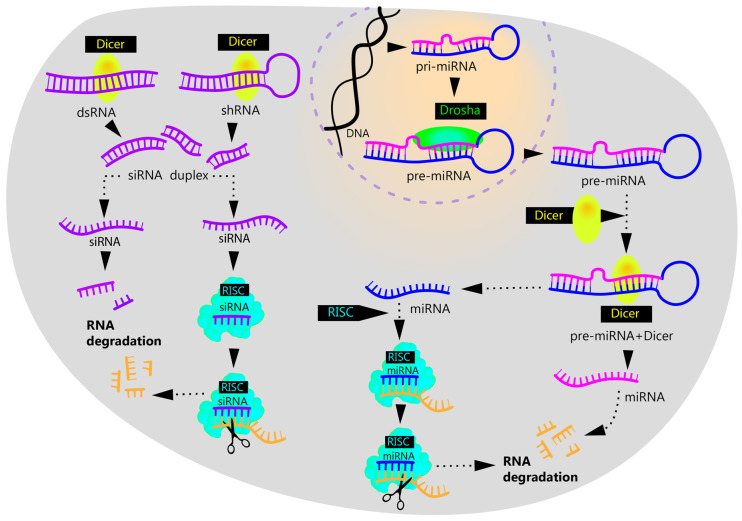
Mechanism of RNA interference. (1) Long double-stranded RNA (dsRNA) or shRNA is recognized by the Dicer protein, which cleaves siRNA into short duplexes of nucleotides. These duplexes split into two single-stranded siRNAs, of which one is subject to degradation, and the other is integrated into the RISC complex. The formed complex specifically binds to the target mRNA, which leads to its cleavage and further degradation. (2) The miRNA precursor is formed on the basis of genomic DNA under the effect of RNA polymerase II, which induces the formation of an intermediate form of pri-miRNA that is cleaved by the Drosha enzyme. The resulting product, pre-miRNA, is transported to the cytoplasm where it is cleaved by the Dicer protein to miRNA, whose mechanism of action is similar to that of siRNA.

Small interfering RNAs (siRNAs) and miRNAs are components of the RNA interference system, which regulates gene expression. SiRNAs are formed from double-stranded RNAs with the Dicer enzyme, which cleaves these molecules into fragments of 20–24 nt in size. Unlike siRNA, miRNA is formed from separate genes in the form of primary RNA molecules (pri-miRNA). These molecules are processed by the Drosha enzyme in the nucleus and then exported to the cytoplasm for further processing by Dicer. As a result of Dicer processing, mature siRNA and miRNA molecules are formed that are then integrated in the RISC complex and, as a component of this complex, bind to mRNA, thus affecting the regulation of expression [87,88]. SiRNA usually binds to mRNA with full complementarity, which leads to its specific degradation. In contrast, miRNAs can bind to mRNAs less strongly. Such binding does not always cause mRNA degradation, but it may hamper the translation process Figure 3. 

Furthermore, small hairpin RNA (shRNA), which acts by the siRNA mechanism and is its precursor, can also be used in some cases. Its major advantage is the stable inhibition of expression, whereas siRNA acts temporarily.

#### Small Interfering RNAs and miRNAs in ALS Treatment

Since 2003, it has become known that siRNAs and shRNAs are capable of selectively suppressing mutant *SOD1* genes in vitro [89]. In 2005, a study on a model of transgenic mice with the human *SOD1*^G93A^ mutation demonstrated that the delivery of siRNA using an adeno-associated virus delays the loss of grip strength in the limb where the injection was made [90]. Injections with the lentiviral vector led to an efficient reduction in *SOD1* expression, an increase in survival by nearly 80% of normal lifespan, and improved survival of motor neurons in the spinal cord and the brainstem [91]. Subsequently, the same results were obtained upon treatment with an adeno-associated vector encoding shRNA [92] and were consistent with the data recorded from rats [93]. Efforts to improve this method have not been undertaken until now. The new drug, developed in 2024 after clinical trials of tofersen, was expected to improve therapeutic effects. Studies on mice showed its greater effectiveness at doses about 3-fold lower than those of antisense oligonucleotides, which opens up new opportunities in the treatment of diseases associated with *SOD1* mutations [94].

MiRNA has been used for the treatment of ALS since 2014. Anti-SOD1 miRNA, delivered to CNS cells of primates and mice via an rAAVrh10 vector, demonstrated its ability to slow down the progression of the disease and extend survival, although it did not significantly alter the disease onset based on the age of peak body weight [95]. In an experiment on mice, the treatment of miRNA SOD1 delivered via a similar virus led to a 50% decrease in the expression of mutant mRNA in the CNS, which extended survival from 137 to 206 days and delayed hind limb paralysis [96].

Studies on RNA interference therapy against the mutant *C9orf72* gene were launched in 2013. During the experiments using siRNA on fibroblasts, researchers managed to reduce the level of *C9orf72* RNA to 30% of controls. With the use of siRNA on fibroblasts, it was possible to reduce the level of *C9orf72* mRNA to 30%, but this decrease did not affect the accumulation of expanded RNA foci in cells [79]. In 2019, a new miRNA-based therapy was carried out, for which an AAV was used as a delivery system. Cell culture studies demonstrated a successful decrease in *C9orf72* transcripts containing nucleotide repeat expansions without affecting normal mRNA levels of this gene. Furthermore, a transcript decrease was also recorded from mice, where the level of suppression ranged from 20 to 40%. However, no behavioral changes were observed in the animals, which necessitates further studies in this direction [97].

Therapy against the M337V mutation in the *TARDBP* gene was carried out by using allele-specific siRNA. In experiments with a neuronal stem cell model, siM9 reduced the total level of the endogenous TDP-43^M337V^ by 40% [98]. In the model of fibroblasts with the G376D mutation, siRNA reduced the amount of aggregates from 60 to 15%, which improved cell viability and prevented oxidative stress development [99].

Mutations in the *FUS* gene occur in only 4% of familial forms, and therefore, genetic methods are not developed for it as actively as for the *SOD1* and *C9orf72* genes. To date, there are no published studies on the use of RNA interference methods aimed at this gene.

##### Clinical Studies

In 2024, the first drug from the RNA interference group, RAG-17, entered phase 1 clinical trials. Previously, the drug showed its effectiveness for the treatment of rats and monkeys: it delayed the disease onset, increased the median lifespan to 215 days, and improved motor function in *SOD1*^G93A^ mutant rats [100]. The publication of the first results of this study is scheduled for 2026.

In 2020, a study was published on the use of miRNA SOD1 for the treatment of two ALS patients, where an adeno-associated virus was also used to deliver it. The first patient had a decrease in SOD1 level in the spinal cord, but the level of this protein in the CSF did not change. It was difficult to draw any conclusions about the efficacy of the therapy, although at 14 months after the initiation of treatment, the patient regained the ability to extend and flex the fingers of the left hand, with other clinical signs and respiratory activity decreased, which is typical of patients with ALS. In the second patient, miRNA therapy did not show any clinical efficacy [101].

### 3.3. CRISPR/Cas9 Method

In 2012, the development of the CRISPR/Cas9 system by Emmanuelle Charpentier and Jennifer Doudna became a revolutionary discovery that allowed scientists worldwide to conduct basic research and make precise changes to DNA [102]. The CRISPR/Cas9 technology for the treatment of neurodegenerative disorders is actively studied on animal models and cell cultures. Some promising studies have already shown the potential of CRISPR/Cas9 to be used for the treatment of various genetic disorders such as Huntington’s disease [103], Alzheimer’s disease [104], Duchenne muscular dystrophy [105], and ALS [106,107].

CRISPR/Cas9 is a genome-editing method that allows for precise changes to DNA by deleting, adding, or modifying its sequences. The application of CRISPR/Cas9 includes several stages:

1. Target selection, i.e., the selection of a specific gene or sequence that needs to be edited.
2. Design of guide RNA (sgRNA), which is an RNA complementary to the selected DNA sequence necessary for directing the Cas protein towards the target for subsequent correction [108].
3. The third stage is to deliver the CRISPR/Cas9 system to the cell. Viral and non-viral vectors can be used for this purpose.
4. Formation of the Cas9–sgRNA complex can be divided into two major stages:
  (1) Binding stage:

The guide RNA includes a permanent component referred to as tracrRNA (trans-activating CRISPR RNA) that provides the formation of a structure capable of binding to the Cas protein and forming the Cas9–sgRNA complex.

(2) Target recognition stage:

The second part of gRNA is a spacer fragment formed by a sequence of 20 nucleotides and required for directing the CRISPR/Cas9 complex to a specific site in the genome referred to as the protospacer. Next to the protospacer, there should be PAM (protospacer-adjacent motif), a short sequence of DNA upon binding to which the cleavage of Cas9 is activated. For Cas9, the PAM sequence usually consists of three nucleotides and has a form of NGG, where N can represent any nucleotide.

5. Cleavage of DNA chains:

After the binding of Cas9–sgRNA to the target DNA sequence, the Cas9 protein creates breaks in both chains, rupturing the bonds in the sugar–phosphate backbone of DNA. Depending on the editing purposes, different proteins from the Cas family can be used. The most common protein is Cas9, which can make double breaks in DNA to introduce new sequences [102]. Moreover, other proteins such as Cas12 and Cas13 can be used instead of Cas9. The Cas12 protein, unlike Cas9, cuts DNA, creating single-stranded breaks at the target’s ends. This can be useful when more precise changes to the genome are required [109]. In turn, Cas13 was developed for RNA editing, which makes it indispensable when necessary to temporarily change the functions of genes [110].

6. The resulting break triggers a reaction aimed at its repair. To correct double-stranded breaks, cells can involve one of two major mechanisms: (1) non-homologous end joining (NHEJ), which can lead to small insertions or deletions at the break site and, as a result, to gene knockout; (2) homology directed repair (HDR), allowing for the accurate repair of the break using a template, which provides an opportunity to make specific changes to the genome or even add new genes.
7. The last stage is an analysis to assess the accuracy of the changes made. For this, the genome or its specific site is sequenced.

#### Use of the CRISPR/Cas9 Method for ALS Treatment

Unlike gene therapy methods based on oligonucleotides, the CRISPR/Cas9 genome-editing technology is an approach that provides the permanent shutdown of a mutant gene’s function.

Since 2017, this method has been actively studied in order to suppress the activity of mutant genes in fibroblasts such as the A272C mutation in the *SOD1* gene and G1566A in the *FUS* gene, which were recorded from two ALS patients, respectively. The CRISPR/Cas9 system was introduced using a plasmid vector. The efficacy of targeting the *SOD1* gene proved to be about 20%, and that for the *FUS* gene was about 1% [21]. Also in 2017, an important experiment was set up where the CRISPR/Cas9 system was introduced by an AAV vector on an ALS model of mice with the G93A-SOD1 mutation. As a result, there was a 3-fold decrease in the expression of mutant *SOD1* in the spinal cord, which led to a delay in the disease onset in the mice by an average of 33 days. Furthermore, the mean survival of the animals also increase by 28–30 days [107]. In 2019, targeting the CRISPR system in the G93ASOD1 transgenic mice led to a decrease in the fluorescence intensity of the SOD1 protein in motor neurons and the inhibition of activated microglia, which provided an improvement in clinical manifestations within four weeks [111]. Similar results were obtained with the intrathecal administration of CRISPR/Cas9. The study showed not only a 40% reduction in immunoreactive SOD1 inclusions but also improvement in neuromuscular function [22]. These achievements confirm the efficacy of the method on this model.

In 2020, several genome-editing studies based on the CRISPR/Cas9 system focused on motor neurons obtained from patients with the mutant *C9orf72* gene. This approach allowed for restoring the normal expression of the *C9orf72* gene and eliminating pathological forms of the protein [112,113]. Furthermore, it was found that the deletion of the minimal promoter located in the exon-1a of the *C9orf72* gene eliminated neuronal degeneration [113].

Following the first successful correction of the *FUS* gene in 2017, [21] new cell-line-based studies have demonstrated that the mutation correction by CRISPR/Cas9 efficiently eliminates DNA ligation defects [114]. As regards the M337V mutation of the *TARDBP* gene, it was successfully corrected in 2019, as was evidenced by the recovery of synaptic plasticity and normalization of BDNF secretion [115]. These data confirm the high efficiency of the CRISPR/Cas9 method, which opens up new opportunities for further research.

### 3.4. Vector-Based Gene Therapy

Most therapeutic agents discussed above in gene therapy are delivered using viral and non-viral vectors. Currently, viral vectors are considered more efficient due to their ability to infect cells and introduce their genetic material into them. However, their significant drawback is the potentially pronounced immune response to their administration.

The main representative among viral vectors to deliver materials to nervous system cells is the adeno-associated vector (AAV vector). Its main distinguishing feature is the lack of the ability to replicate independently, and, as a result, it needs the presence of adenovirus or other viruses for successful reproduction [116,117]. AAV vectors can infect both dividing and non-dividing cells. The lack of a significant immune response to the introduction of the AAV vector makes it one of the promising tools for gene therapy among all viral vectors. This also opens up a wide range of opportunities for repeated injections, which is an important aspect for the treatment of chronic diseases and genetic disorders [118]. In most cases, after infection, AAV vectors form their own plasmids that can be located in the nucleus in the form of episomes, which are DNA molecules that cannot be integrated into chromosomes and alter the expression of the target gene for a short time. This, in turn, reduces the risk of mutations and carcinogenesis.

**Figure 4:**
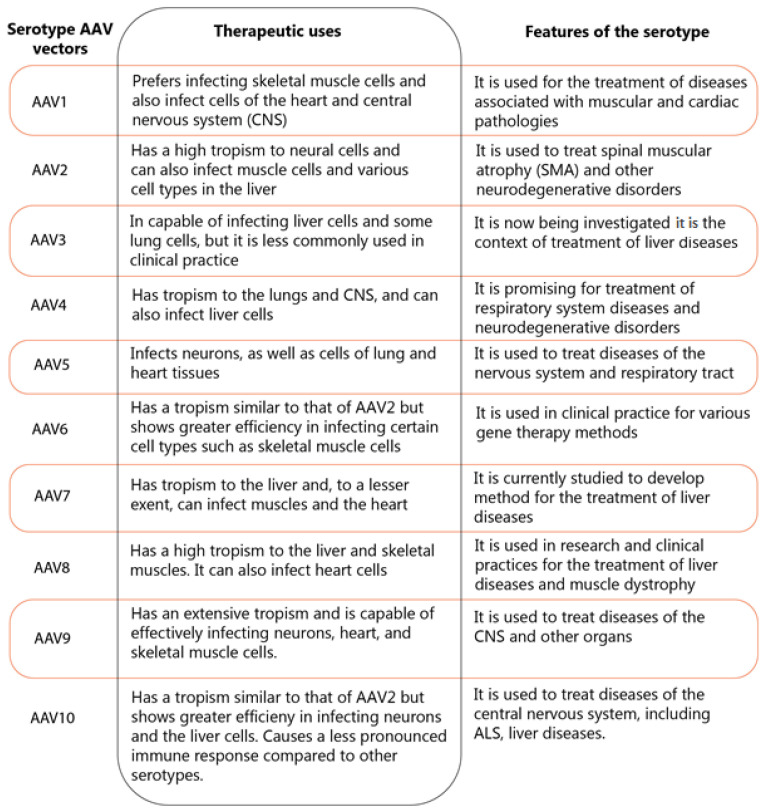
Features and therapeutic uses of AAV vector serotypes.

Different serotypes of AAV vectors exhibit their own unique characteristics of tropism and diffusion ability. These features make them efficient for use in various therapeutic approaches Figure 4 [119,120,121,122,123,124,125,126,127]:

Despite the relative safety and efficiency of delivery by AAVs, researchers are attempting to minimize their use because the administration of glucocorticosteroids is still required for patients in some cases [96], which significantly complicates treatment. For this reason, non-viral vector-based delivery methods are being sought that include the administration of lipid nanoparticles, magnetic nanoparticles, and electroporation methods.

Despite their limitations, their effectiveness and high demand for the delivery of various therapeutic components, including miRNA, siRNA, CRISPR/Cas9 components, make them indispensable in the development of new therapeutic approaches to ALS. The use of viral vectors is certainly possible without the use of additional technologies, but such studies are currently few [128].

## 4. Conclusions

Gene therapy is a unique tool that allows us to correct various genetic defects and significantly improve symptoms. We have covered gene therapy methods that include antisense oligonucleotides, RNA interference methods (siRNA and microRNA), and the genome-editing method (CRISPR/Cas9). Among them, human-genome-editing methods remain limited to this day due to ethical considerations, while methods that affect gene expression are being studied more actively, and although their effects are temporary, they certainly show impressive results in animal models of ALS. However, these methods are not always effective in clinical trials. And complete recovery cannot be achieved in either animals or humans.

This highlights the need for new research, as well as the active development of other methods of drug support for patients aimed at the following pathogenetic mechanisms: glutamate excitotoxicity, oxidative stress, mitochondrial dysfunction, and protein aggregation. It is likely that combined methods will help improve the clinical symptoms of patients and help increase life expectancy.
